# Overcoming Intra-Articular Delivery Barriers in Cartilage Regeneration: Emerging Controlled-Release Strategies

**DOI:** 10.3390/biom16101457

**Published:** 2026-10-07

**Authors:** Fajer Yousef, Muskan Alad, Laura M. Epure, Angelina Lui, Michael P. Grant, Nicoletta Eliopoulos, John Antoniou, Fackson Mwale

**Affiliations:** 1 Department of Surgery, Division of Surgical and Interventional Sciences, McGill University, Montreal, QC H3G 1A4, Canada; fajer.yousef@mail.mcgill.ca (F.Y.); laura.epure@mcgill.ca (L.M.E.); nicoletta.eliopoulos@mcgill.ca (N.E.); janton@orl.mcgill.ca (J.A.); 2 Orthopaedic Research Laboratory, Lady Davis Institute for Medical Research, SMBD-Jewish General Hospital, 3755 Chemin de la Côte-Sainte-Catherine, Montreal, QC H3T 1E2, Canada; muskan.alad@mail.mcgill.ca (M.A.); angelina.lui@mail.mcgill.ca (A.L.); michael.grant@affiliate.mcgill.ca (M.P.G.)

**Keywords:** articular cartilage, intra-articular drug delivery, controlled release, cartilage penetration, nanoparticles, nanodiamonds, osteoarthritis, regenerative medicine

## Abstract

Articular cartilage has a limited capacity for self-repair due to its avascular nature, dense extracellular matrix, and low cellularity. Current treatments for cartilage injury and osteoarthritis (OA) often provide temporary symptom relief but do not restore native tissue structure or function. Intra-articular (IA) administration enables localized delivery of therapeutic agents; however, its effectiveness is limited by rapid clearance from the joint, poor cartilage penetration, and insufficient tissue retention. Controlled-release systems, including scaffolds, hydrogels, nanoparticles, and stimuli-responsive platforms, have been developed to address these barriers by prolonging local therapeutic exposure and enabling sustained or targeted delivery. Preclinical studies have demonstrated the potential of these systems to reduce inflammation and support cartilage protection and repair. However, clinical evidence remains limited, and further studies are required to determine whether these delivery advantages translate into sustained disease-modifying or regenerative outcomes in patients. This review discusses the biological barriers that limit effective IA delivery and summarizes recent advances in controlled-release strategies, with particular attention to their ability to improve joint retention, cartilage penetration, and sustained therapeutic exposure. The advantages and limitations of the different delivery platforms are critically discussed, together with the translational challenges that currently limit their progression from preclinical studies to clinical application.

## 1. Introduction

Articular cartilage (AC) is a highly specialized connective tissue that provides a smooth, low-friction articulating surface while distributing mechanical loads across synovial joints. However, its avascular, alymphatic, and aneural nature severely restricts nutrient delivery, cellular recruitment, and tissue repair following injury [1,2,3]. Unlike most tissues, AC relies almost entirely on diffusion from synovial fluid for nutrient supply and metabolic waste removal, a relatively inefficient process that becomes further compromised after injury. In addition, chondrocytes, the sole resident cells within cartilage, possess limited proliferative and migratory capacity, restricting their ability to restore the damaged extracellular matrix (ECM). Consequently, even small cartilage defects often progress to chronic degeneration, pain, impaired joint function, and ultimately osteoarthritis (OA) [4,5,6], making cartilage regeneration one of the greatest challenges in regenerative medicine [1,7,8].

To overcome the limited intrinsic healing capacity of cartilage, numerous therapeutic strategies have been developed, ranging from symptomatic approaches that alleviate pain and improve joint function to disease-modifying OA drugs aimed at slowing or preventing structural deterioration, and regenerative therapies designed to restore damaged cartilage through new ECM formation and tissue repair [9,10].

Regenerative approaches include stem cell therapy, growth factor delivery, gene therapy, and other biologic approaches [5,7,9,11]. While these therapies have demonstrated encouraging preclinical and early clinical outcomes, their success depends not only on the therapeutic agent itself but also on its ability to reach, penetrate, and remain within the target tissue [9,10,11,12,13]. Following intra-articular (IA) administration, many biologics, proteins, nucleic acids, and small molecules are rapidly eliminated through synovial fluid turnover and lymphatic drainage, resulting in short residence times and insufficient exposure at the site of injury [14,15,16,17,18].

These biological and pharmacokinetic challenges have shifted attention toward controlled-release drug delivery systems designed to improve local retention and sustained therapeutic activity within the joint. By protecting bioactive agents from degradation and providing localized, prolonged release, these delivery platforms can extend IA residence time, reduce injection frequency, maintain effective drug concentrations, and ultimately enhance cartilage regeneration [7,10,19,20,21,22]. A variety of delivery systems, including scaffolds, hydrogels, nanoparticles, and stimuli-responsive platforms, are currently being investigated to improve the efficacy of regenerative therapies for cartilage repair [19,20]. Among these, nanoparticle-based delivery systems have attracted particular interest because of their ability to encapsulate diverse therapeutic cargos, facilitate controlled release, improve tissue penetration, and enable surface functionalization for targeted delivery.

Recent reviews have described advances in IA drug delivery, cartilage-targeting strategies, and controlled-release systems for OA [14,18,19,21,22]. This review considers these developments in relation to the key barriers that limit therapeutic exposure within articular cartilage, including rapid joint clearance, poor tissue retention and penetration, therapeutic degradation, and insufficient duration of local activity. Rather than classifying delivery platforms solely by material type, we assess how scaffolds, hydrogels, nanoparticles, and emerging responsive systems address each barrier and critically evaluate the evidence for cartilage protection, disease modification, and regeneration. We also highlight the translational limitations that continue to separate promising preclinical delivery systems from clinical application.

For the literature selection, relevant studies were identified through PubMed searches conducted from January 2005 to August 2026 using combinations of terms related to articular cartilage, osteoarthritis, intra-articular drug delivery, controlled release, sustained release, cartilage targeting, hydrogels, scaffolds, nanoparticles, nanodiamonds, and stimuli-responsive delivery systems. Priority was given to recent peer-reviewed studies addressing IA retention, cartilage penetration, controlled or sustained therapeutic release, and cartilage-related outcomes. Original experimental studies were preferentially cited when discussing specific delivery systems and therapeutic effects, while relevant reviews and landmark studies were included to provide background and broader context. Studies in which the joint was not the site of administration were included only where they provide the best available toxicological or biodistribution data and are identified as such in the text. Because no ND study has yet reported cartilage penetration or IA pharmacokinetics, the ND section draws on the wider ND biocompatibility literature and states this limitation explicitly. The literature selection was narrative rather than systematic and was focused on studies most relevant to the scope of this review.

## 2. Biological Barriers to Cartilage Regeneration

The biomechanical properties of AC are largely determined by its ECM, which accounts for approximately 95% of the tissue volume and is composed primarily of water, type II collagen, and proteoglycans [1,3,23,24]. Type II collagen forms a dense fibrillar network that provides tensile strength and maintains tissue architecture, while proteoglycans, predominantly aggrecan, contain negatively charged glycosaminoglycan (GAG) chains that attract water and generate the osmotic pressure required to resist compressive forces [20,24,25]. Together, these matrix components enable cartilage to withstand repetitive mechanical loading while maintaining flexibility and resilience [20,24].

**Figure 1:**
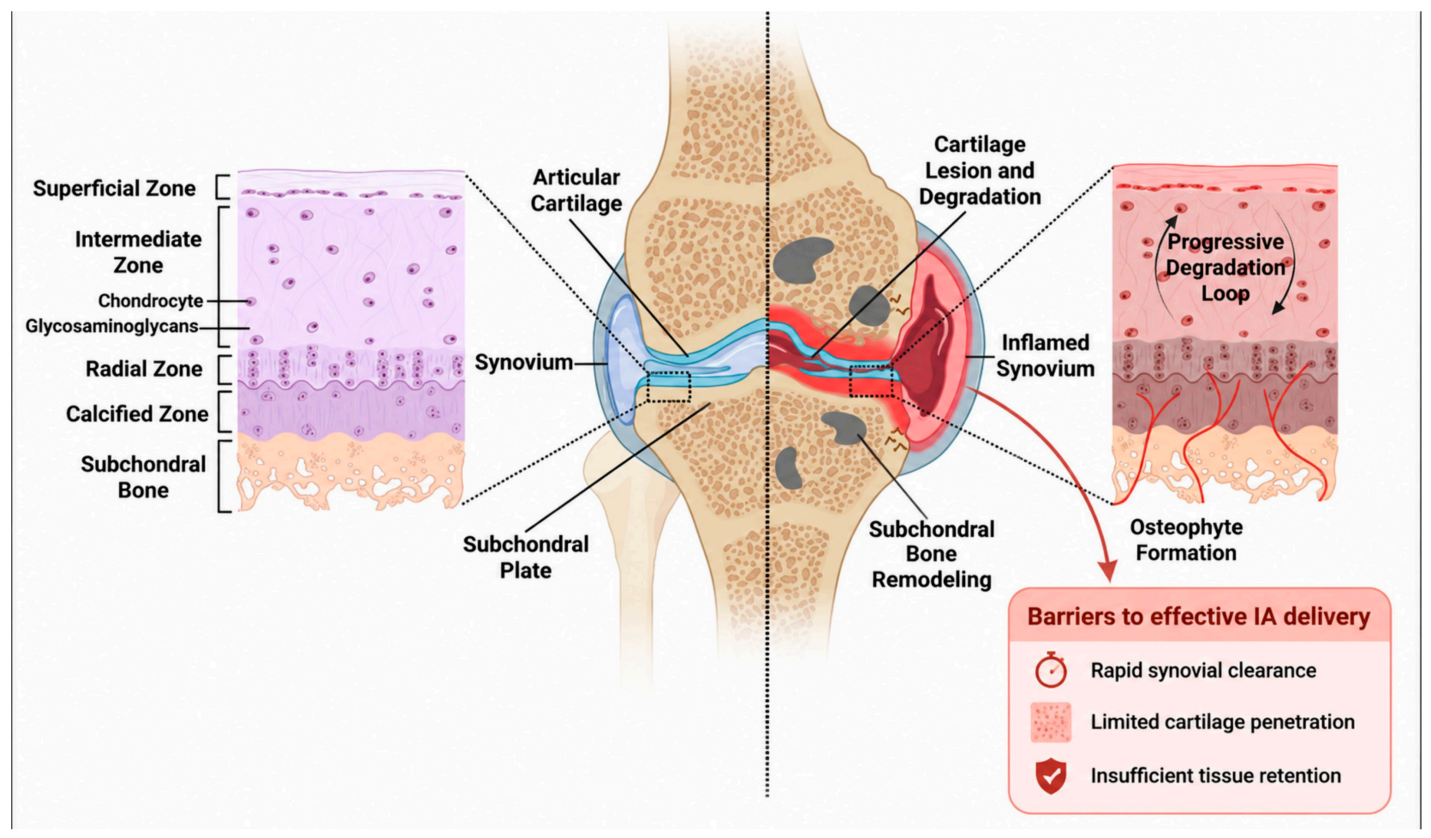
Schematic representation of osteoarthritis pathogenesis and major barriers to intra-articular drug delivery. The diagram illustrates structural and biological changes occurring across joint tissues during osteoarthritis. Progressive degradation of the articular cartilage, beginning at the superficial surface, contributes to cartilage lesions and extracellular matrix loss. The synovium becomes inflamed, while the subchondral bone undergoes remodeling and osteophyte formation. These pathological changes occur within an intra-articular environment in which rapid synovial clearance, limited cartilage penetration, and insufficient tissue retention can restrict therapeutic exposure within articular cartilage.

Proteoglycan content is not uniform through the tissue depth: fixed charge density and GAG concentration are lowest in the superficial zone and increase through the middle (intermediate) zone to the deep (radial) zone, whereas cell density follows the opposite gradient (Figure 1) [1,3,26]. This zonal organization matters for drug delivery. The superficial zone, which an injected therapeutic encounters first, is the collagen-rich and GAG-poor layer, and the increasing fixed negative charge with depth is what a cationic carrier exploits for electrostatic penetration and retention [27,28]. The dense, highly organized, and negatively charged ECM therefore creates a diffusion barrier that limits the entry of many drugs, biologics, and regenerative molecules into the deeper layers where most of the aggrecan resides [18,25,28]. Under physiological conditions, particles larger than a few tens of nanometres are largely excluded from full-thickness penetration, and neutral or anionic species that do enter are cleared rapidly; by contrast, cationic carriers below approximately 10 nm penetrate full-thickness cartilage within hours, and 38 nm particles bearing a type II collagen-binding peptide have entered and been retained in the matrix [28,29,30]. However, carrier matrix interactions must be appropriately balanced, as increased binding can enhance tissue retention while potentially restrict diffusion into deeper cartilage regions [25,31]. Cartilage thickness can further influence transport by increasing the diffusion distance required to reach deeper tissue regions [32]. Disease-associated changes in proteoglycan content and matrix organization may alter cartilage permeability and the interactions between therapeutic carriers and ECM [25,27].

Chondrocytes, the sole resident cells of AC, are responsible for maintaining ECM homeostasis through the continuous synthesis and turnover of matrix components [27,33,34]. Following injury, they attempt to restore damaged tissue by increasing matrix production; however, their limited proliferative and migratory capacity, combined with their sparse distribution within the dense ECM, severely restricts their regenerative potential [7,27,34]. Additionally, mechanical overload, oxidative stress, inflammatory cytokines, and matrix degradation products can further disrupt chondrocyte homeostasis, shifting cells toward a catabolic phenotype characterized by increased production of matrix metalloproteinases (MMPs), aggrecanases (ADAMTS), and pro-inflammatory mediators, thereby accelerating cartilage degeneration [34,35]. Consequently, damaged cartilage rarely regains its native architecture, resulting in progressive structural deterioration and eventual OA if left untreated [5,27].

The absence of blood vessels, lymphatics, and nerves is one of the defining features of AC and a major reason for its diminished healing capacity [27,36]. Nutrient delivery and waste removal rely entirely on diffusion from synovial fluid, an inherently inefficient process that becomes even less effective following injury [27,37]. Unlike vascularized tissues, cartilage cannot readily recruit inflammatory cells, progenitor cells, or circulating growth factors to sites of injury, further restricting tissue repair [5,27]. In addition, the dense ECM limits the penetration of reparative cells and therapeutic agents into deeper cartilage layers [38,39]. As a result, cartilage defects frequently remain unrepaired and progressively worsen over time [11,27]. Additionally, the inflammatory microenvironment present in OA joints exposes therapeutic agents to proteolytic enzymes, reactive oxygen species (ROS), and inflammatory cytokines that may reduce their stability and biological activity before they reach their target cells and exert sustained therapeutic effects [27,35].

Mechanical trauma, aging, obesity, joint instability, and chronic inflammation disrupt cartilage homeostasis and initiate a cascade of degenerative events that frequently result in OA [35,40]. Activated chondrocytes release inflammatory cytokines together with matrix-degrading enzymes, including MMPs and aggrecanases, leading to progressive degradation of collagen and proteoglycans (Figure 1) [27,35]. Fragments generated during ECM degradation function as damage-associated molecular patterns (DAMPs), amplifying inflammatory signaling within the joint [41,42]. Disease progression subsequently extends beyond cartilage to involve the synovium and subchondral bone where persistent production of inflammatory cytokines, including interleukin-1β (IL-1β) and tumor necrosis factor-α (TNF-α), together with abnormal bone remodeling, establishes a self-perpetuating cycle of inflammation and tissue destruction [35,43].

Furthermore, continuous synovial fluid turnover and lymphatic drainage rapidly clear many therapeutics from the joint space, resulting in short IA residence time and reduced treatment efficacy [44,45]. To overcome these limitations, controlled-release delivery platforms have been developed to protect therapeutic agents, prolong IA retention, and provide sustained local drug release, thereby enhancing the therapeutic potential of regenerative therapies for cartilage repair and OA [46,47].

## 3. Controlled-Release Delivery Platforms for Cartilage Regeneration

Pharmacokinetics is the main challenge of many IA therapies [14,21,38,45]. Small molecules, proteins, cytokines, and growth factors are often cleared within hours following injection, while even larger molecules such as hyaluronic acid demonstrate relatively short IA half-lives [17,38]. Albumin, for example, may be cleared from the synovial cavity within approximately 13 h, whereas hyaluronic acid typically exhibits a residence time of approximately 26 h depending on molecular weight and formulation [15,48]. Consequently, therapeutic concentrations are often difficult to maintain, necessitating repeated injections and increasing treatment burden [38]. Controlled-release delivery systems have been developed to overcome the pharmacokinetic limitations of conventional IA therapies by improving local drug retention, protecting therapeutic agents from degradation, and enabling sustained release within the joint [18,19,47,49]. Among the various platforms investigated, scaffolds, hydrogels, nanoparticles, and hybrid systems each offer distinct advantages depending on the therapeutic application, drug cargo, and desired release profile [19,47]. Representative controlled-release and cartilage-targeting strategies are compared in Table 1 according to the principal IA delivery barrier addressed, retention or release characteristics, cartilage penetration or targeting, therapeutic outcomes, and key limitations.

### 3.1. Scaffolds

Scaffolds serve as three-dimensional biomaterial depots that localize therapeutic agents at cartilage defect sites while providing structural support for tissue repair. By mimicking key features of the native ECM, these constructs can present growth factors, anti-inflammatory drugs, regenerative cells, or other bioactive cargos in a localized and sustained manner, thereby supporting cell attachment, proliferation, differentiation, and matrix deposition [22,27,33,36]. Scaffold performance depends strongly on material composition and architecture. Natural biomaterials, including collagen, gelatin, fibrin, hyaluronic acid, alginate, and chitosan, offer inherent bioactivity and biocompatibility, whereas synthetic polymers such as poly(lactic-co-glycolic acid) (PLGA), polyethylene glycol (PEG), and polycaprolactone (PCL) provide greater control over porosity, mechanical properties, degradation kinetics, and release behavior [33,36,62]. However, natural scaffolds generally have lower mechanical strength and less predictable degradation, while synthetic systems often require surface modification or bioactive functionalization to improve cellular interactions and therapeutic presentation [33,36,47].

An important advantage of scaffold-based systems is their ability to localize therapeutic agents within cartilage defects and release them gradually through diffusion, matrix degradation, or both. This prolonged local delivery supports sustained biological activity while reducing the need for repeated IA injections [21,22]. In experimental models, scaffolds incorporating growth factors such as transforming growth factor-β3 (TGF-β3) or seeded with mesenchymal stromal cells (MSCs) and chondrocytes have demonstrated enhanced ECM formation, improved histological repair, and increased hyaline-like cartilage formation [33,50,62,63]. Several scaffold technologies have reached clinical application, and they are the only platforms discussed in this review with human evidence of structural repair. Matrix-induced autologous chondrocyte implantation (MACI^®^) employs a collagen membrane seeded with autologous chondrocytes; in randomized comparison with microfracture it produced better structural repair at 12 months and better clinical outcomes at 5 years in focal defects of the knee, although it is a surgical cell-implantation procedure rather than a controlled-release product, and its indication is the focal defect rather than OA [64,65]. Similarly, Cartistem^®^, which combines umbilical cord blood-derived MSCs with a hyaluronic acid scaffold, has demonstrated encouraging structural and clinical outcomes in a small trial with 7 years of follow-up and is approved for clinical use in South Korea [66].

Despite these advances, scaffold-based therapies remain limited by the need for surgical implantation, challenges in achieving complete integration with surrounding cartilage, and the difficulty of matching scaffold degradation to the rate of new tissue formation. Manufacturing complexity, patient-specific defect characteristics, and variability in the biological response further influence clinical outcomes [27,36,62]. Current research is focused on multifunctional scaffold systems that combine structural support with advanced drug delivery strategies. Composite scaffolds incorporating nanoparticles, hydrogels, or bioactive surface modifications offer improved control over release kinetics while enhancing mechanical performance and biological activity. These platforms may improve control over therapeutic release; however, their increasing formulation complexity also introduces challenges related to manufacturing reproducibility, long-term safety, and clinical translation [19,22].

### 3.2. Hydrogels

Hydrogels are widely investigated as controlled-release platforms for AC regeneration because their hydrated, three-dimensional polymer networks can encapsulate therapeutic agents while providing a localized depot within the joint [21,22]. They can be formed from natural polymers, including hyaluronic acid, collagen, gelatin, chitosan, and alginate, or synthetic materials such as PEG, polyvinyl alcohol (PVA), and PLGA-based copolymers. Material composition directly influences their performance as delivery systems. Natural polymers offer high biocompatibility and intrinsic interactions with cells and ECM components but may show greater variability in mechanical stability and degradation, whereas synthetic polymers provide greater control over network architecture, crosslinking density, swelling, mechanical properties, and degradation kinetics. Hybrid systems can combine these characteristics to improve both biological compatibility and control over drug release [19,22,67].

Following IA administration, injectable hydrogels can form a localized reservoir that reduces rapid synovial clearance and prolongs therapeutic exposure. Cargo may be physically entrapped within the polymer network, chemically conjugated to the matrix, or incorporated through secondary carriers such as nanoparticles or microspheres. Release can occur through diffusion, hydrogel swelling or degradation, cleavage of drug–matrix interactions, or combinations of these mechanisms. Accordingly, polymer composition, mesh size, crosslinking density, charge, and degradation rate can be tailored to regulate cargo loading, retention, and release kinetics [19,22,67].

One of the principal advantages of hydrogels is their ability to encapsulate a wide range of therapeutic agents, including anti-inflammatory drugs, corticosteroids, growth factors, biologics, extracellular vesicles, and stem cells [21,22]. In preclinical models, hydrogel-based delivery of TGF-β, insulin-like growth factor-1 (IGF-1), interleukin-1β (IL-1β) antagonists, and chondrocyte- or MSC-derived exosomes has reduced synovial inflammation and preserved cartilage structure [51,68,69,70]. For example, a thermosensitive hydrogel loaded with chondrocyte-derived exosomes shifted synovial macrophages toward an M2 phenotype and reduced cartilage loss in a rat OA model [51]. However, these findings represent chondroprotective outcomes in preclinical models rather than direct evidence of cartilage regeneration.

Injectability provides an additional delivery advantage, allowing hydrogels to be administered using minimally invasive techniques and conform to irregular cartilage defects without open surgical implantation, making them particularly attractive for early OA and focal cartilage defects [19,21,22]. Recent advances have led to the development of stimuli-responsive hydrogels capable of releasing therapeutic agents in response to environmental triggers such as pH, enzymatic activity, temperature, ROS, or mechanical loading [19,52,71]. Such systems provide greater spatial and temporal control over cargo release and may reduce systemic exposure and off-target effects [19,22,71].

Despite these advantages, several barriers limit hydrogel-based delivery. Prolonged joint retention does not necessarily ensure effective cartilage penetration: the hydrogel itself generally remains at the injection or defect site, while the released therapeutic agent or secondary carrier must still traverse the dense cartilage ECM to reach chondrocytes in deeper regions [19,22]. Hydrogels may also have difficulty reproducing the load-bearing properties of native cartilage and maintaining predictable degradation and release over prolonged periods. Premature drug leakage, burst release, and insufficient uniform distribution within the joint cavity also continue to remain additional challenges [22,71]. Clinically used hydrogel-based IA products are primarily hyaluronic acid viscosupplements, which provide symptomatic treatment rather than established cartilage regeneration. Despite clinical effects that may persist for months, most injected HA formulations have relatively short IA residence times, generally measured in hours to days [72]. Current research is focused on multifunctional hydrogel systems that incorporate nanoparticles, microspheres, bioactive molecules, or engineered cells to improve mechanical stability and achieve more precise control over therapeutic release [21]. Although these hybrid platforms may improve sustained IA delivery; clinical translation will depend on reproducible release kinetics, mechanical stability, long-term safety, and demonstration of therapeutic benefit beyond preclinical models [19,21,22,71].

### 3.3. Nanoparticles

**Figure 2:**
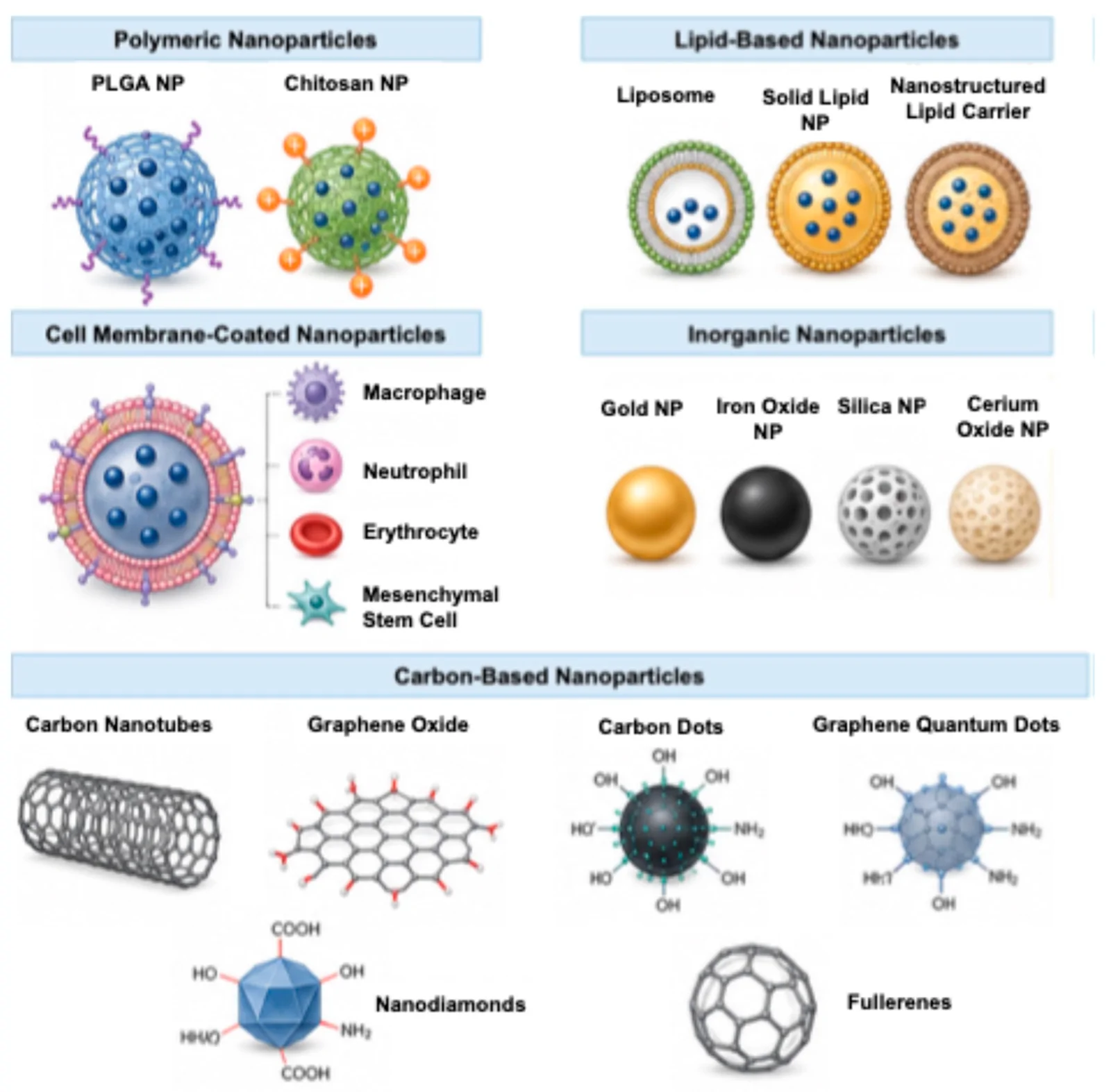
Classification and surface functionalization of nanoparticles for biomedical applications. Nanoparticles are classified into organic, inorganic, and carbon-based systems. Organic carriers, including polymeric and lipid-based nanoparticles, are commonly used for therapeutic encapsulation and sustained release, whereas inorganic nanoparticles may provide imaging, targeting, or catalytic functions. Surface functionalization, such as carboxylation, can modify charge, enable ligand attachment, and influence cartilage penetration and retention.

Nanoparticle-based delivery systems have emerged as promising approaches for enhancing IA drug delivery due to their ability to encapsulate therapeutic agents, improve tissue penetration, and provide sustained, localized drug release [14,22,71,73]. Their small size, high surface-area-to-volume ratio, and tunable physicochemical properties enable efficient delivery of anti-inflammatory drugs, growth factors, proteins, nucleic acids, and other bioactive molecules [21,73]. Nanoparticles are conventionally defined within the 1 to 100 nm size range, although the term nanoparticle is frequently used more broadly in drug-delivery research to include larger submicrometric systems. Nanoparticle-based delivery systems encompass a diverse range of materials and can be broadly classified according to their core composition, including organic nanocarriers such as polymeric and lipid-based systems, and inorganic and carbon-based nanomaterials. In addition, nanoparticles from different material classes can be modified using biomimetic or surface-engineering strategies, including cell membrane coating, to improve cartilage targeting, joint retention, cellular uptake, or responsiveness to disease-specific stimuli [47,74,75,76]. These categories are therefore not necessarily mutually exclusive, as surface modifications can be applied across different nanoparticle cores. The major nanoparticle classes and representative surface-engineering strategies discussed in this section are summarized in Figure 2.

#### 3.3.1. Organic Nanoparticles

Polymeric Nanoparticles

Polymeric nanoparticles are among the most extensively studied systems for cartilage repair because of their biocompatibility, biodegradability, and versatility [77]. Materials such as PLGA, PEG, and chitosan have been widely used to encapsulate anti-inflammatory agents, growth factors, and biologics while enabling sustained drug release [77,78]. At the 100–300 nm sizes typical of many nanoparticle systems, these carriers are generally too large for efficient full-thickness cartilage penetration, while their IA retention remains strongly dependent on particle size, formulation, and joint inflammatory status [28,29,79]. PLGA nanoparticles releasing resveratrol, for example, attenuated cartilage degeneration and inflammatory markers in a rat OA model, a chondroprotective outcome [53]. However, degradation of PLGA generates acidic by-products that may contribute to local inflammation if degradation rates are not carefully controlled [80].

Lipid-Based Nanoparticles

Lipid-based nanoparticles, including liposomes, solid lipid nanoparticles, and nanostructured lipid carriers, offer high biocompatibility and the ability to encapsulate both hydrophilic and hydrophobic therapeutics [21,22,81]. Their lipid-based structure facilitates interaction with cellular membranes and can enhance drug uptake while reducing systemic exposure. Lipid-based systems have been investigated for the IA delivery of anti-inflammatory agents, including liposomal formulations of dexamethasone and diclofenac, as well as celecoxib-containing liposomes combined with hyaluronic acid, with reductions in inflammation and pain behavior in rodent and rabbit OA models [82,83,84]. More recently, IA lipid nanoparticles have also been investigated for localized mRNA delivery, including FGF18 mRNA as an anabolic strategy for OA [54]. Liposomes of 100 nm or more do not penetrate cartilage and act by prolonging release in the synovial space; their relatively limited stability and short IA residence remain important challenges for clinical translation [21,22,81].

#### 3.3.2. Inorganic Nanoparticles

Inorganic nanoparticles, including gold, silica, iron oxide, manganese dioxide, and cerium-based nanoparticles, possess unique optical, magnetic, and catalytic properties that enable both therapeutic delivery and diagnostic applications [22,74,75]. For example, cationic manganese dioxide nanoparticles smaller than 20 nm have been shown to penetrate cartilage explants, scavenge hydrogen peroxide, prevent IL-1β-induced GAG loss, and remain approximately 75% retained in rat knees after one week, thereby combining cartilage penetration, joint retention, and protection against oxidative stress [55]. Similar ROS-scavenging activity has also been reported for ceria and other catalytic oxide nanoparticles [85]. Despite these promising findings, concerns regarding long-term accumulation, biodegradability, and potential toxicity continue to limit their clinical application [21,22,75].

Carbon-Based Nanoparticles

Carbon-based nanomaterials comprise a diverse group of materials, including graphene and its derivatives, carbon nanotubes, carbon dots, graphene quantum dots, fullerenes, and nanodiamonds (NDs), with distinct structural and physicochemical properties that influence their biological behavior and potential biomedical applications [86,87]. Their high surface area and modifiable surface chemistry can support the loading and delivery of drugs, proteins, peptides, and nucleic acids [86,87]. However, biocompatibility should be considered individually for each carbon nanomaterial, as biological responses and toxicity can vary according to particle structure and dimensions, surface chemistry and functionalization, oxidation state, purity, and residual contaminants [86,87]. Therefore, the safety profile of one carbon-based nanomaterial cannot be generalized to the broader class. In OA and cartilage-related applications, selected carbon nanomaterials have been investigated as therapeutic carriers or bioactive materials, although the extent of evidence differs considerably among material types [88].

For example, long, rigid multi-walled carbon nanotubes introduced into the mouse peritoneum produce asbestos-like, length-dependent inflammation and granuloma formation, whereas short or tangled carbon nanotubes elicited substantially less inflammation [89]. These findings raise safety considerations for the use of fibrous carbon nanomaterials in IA applications, for which joint specific safety data remain limited. Graphene-family materials can induce oxidative stress, membrane damage, and inflammatory responses, with toxicity influenced by factors including lateral size, surface structure, functionalization, and oxidation state [90]. Carbon dots cytotoxicity is similarly dependent on surface functionalization and charge, with cationic polyethyleneimine-functionalized carbon dots exhibiting greater cellular uptake and toxicity than neutral PEGylated formulations [91]. Bioconjugated carbon dots have been used to deliver TNF-α siRNA to mesenchymal stromal cells, resulting in suppression of inflammatory signaling and promotion of chondrogenic differentiation in preclinical studies [57]. Other carbon nanomaterials, including graphene derivatives, carbon dots, and fullerenes, have been investigated for antioxidant or anti-inflammatory properties; however, their cartilage-specific evidence and safety profiles remain dependent on the individual material and formulation [86,87]. Water-soluble C60 fullerene has demonstrated chondroprotective effects in OA models, suppressing IL-1β-induced MMP production, and apoptosis in human chondrocytes and reducing cartilage degeneration after IA injection in rabbit OA models [92]. NDs, which consist predominantly of sp3-hybridized carbon, possess distinct physicochemical and surface properties that enable surface functionalization and therapeutic loading, supporting their investigation as drug-delivery carriers [88,93,94,95].

Nanodiamonds as Emerging Controlled Release Carriers

Among carbon-based nanoparticles, NDs have been investigated as controlled-release carriers because of their high surface-area-to-volume ratio, modifiable surface chemistry, chemical stability, and capacity for therapeutic loading [93,94,96,97,98]. Reported ND biocompatibility varies with physicochemical characteristics and surface modification and should therefore be evaluated in the context of the specific formulation [94,97,99]. NDs can be functionalized with a range of therapeutic molecules, enabling efficient drug loading and controlled release through different surface interactions and conjugation strategies [96,97,98]. These characteristics have generated interest in NDs as potential therapeutic delivery; however, cartilage-specific evidence remains limited [96,98].

**Figure 3:**
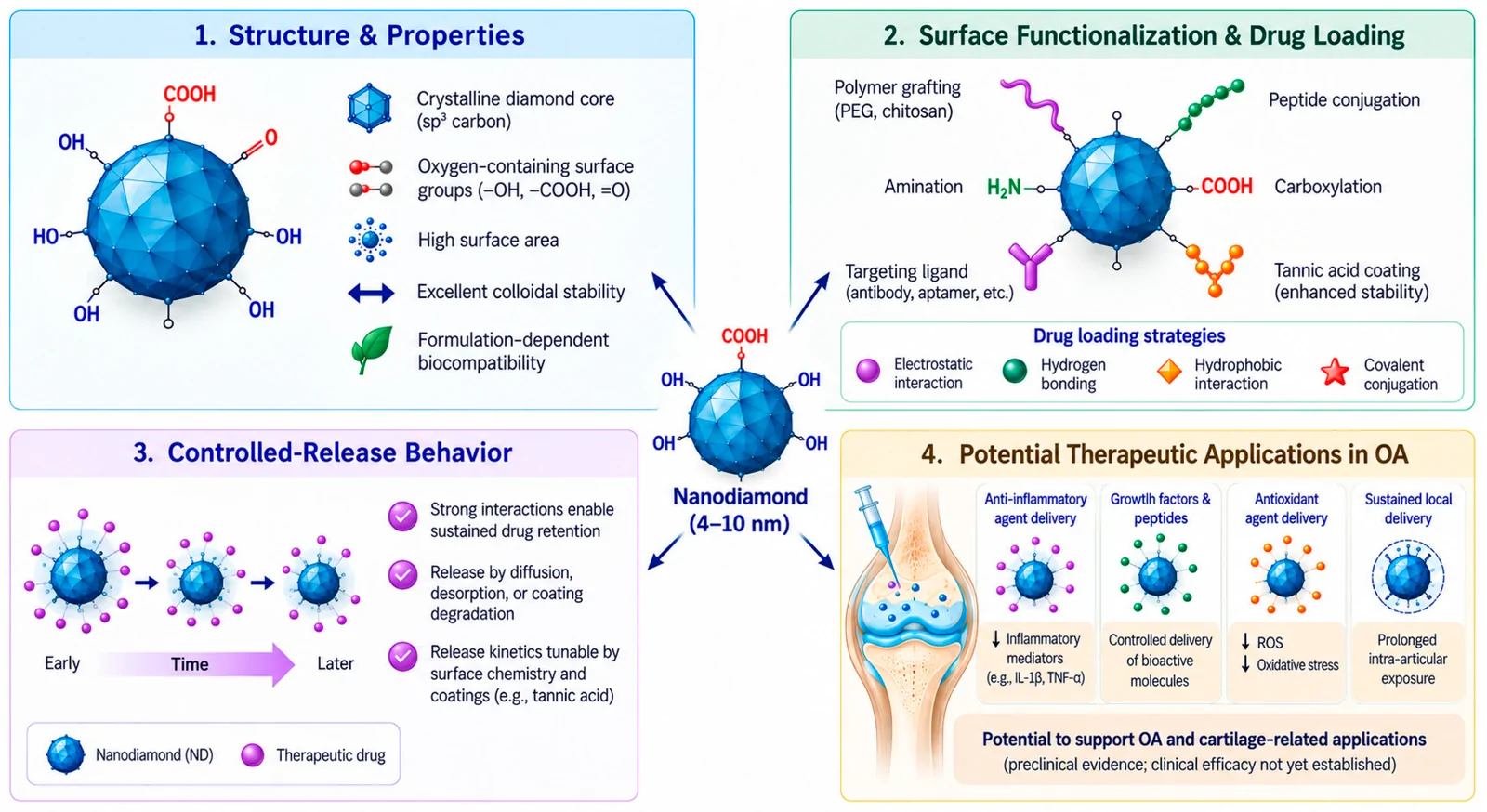
Nanodiamonds (NDs) as emerging controlled-release platforms. The schematic illustrates physicochemical properties of NDs relevant to therapeutic delivery, including modifiable surface chemistry, drug-loading capacity, and controlled-release potential. NDs have been investigated for the delivery of anti-inflammatory agents, growth factors, peptides, and other bioactive molecules in preclinical and regenerative medicine applications. Although these properties support their potential use in OA and cartilage-related therapy, direct evidence for ND-mediated articular cartilage regeneration remains limited.

NDs are carbon-based nanoparticles composed of a crystalline diamond core surrounded by a surface enriched with oxygen-containing functional groups, including hydroxyl, carboxyl, and carbonyl moieties (Figure 3) [93,95,97]. Typically measuring 4–10 nm in diameter, they possess surface functional groups that facilitate conjugation with drugs, proteins, peptides, nucleic acids, and other bioactive molecules while maintaining physicochemical stability [94,96,97,99]. These characteristics support their investigation as controlled-release carriers; however, their suitability for IA delivery requires evaluation in cartilage-specific models, particularly with respect to tissue penetration, retention, clearance, and long-term safety.

Surface Functionalization and Drug Loading

One of the defining characteristics of NDs is their adaptable surface chemistry. Surface functional groups enable chemical modification through oxidation, carboxylation, amination, polymer grafting, peptide conjugation, or adsorption of bioactive coatings (Figure 3) [93,94,95,97,98]. These modifications can improve colloidal stability, regulate surface charge, and enhance drug-loading capacity [95,97,98]. Therapeutic molecules can be loaded onto functionalized ND surfaces through electrostatic interactions, hydrogen bonding, hydrophobic interactions, physical adsorption, or covalent conjugation [100]. Consequently, NDs have been investigated as carrier for a broad range of therapeutics, including drugs, growth factors, peptides, proteins, and nucleic acids [93,97,98]. Surface modification can also be used to regulate interactions between NDs and their therapeutic cargo. Polyphenolic coatings, including tannic acid, have been investigated as a means of modifying surface interactions and controlling therapeutic release [101,102]. Such approaches illustrate the potential of ND surface engineering to tailor drug-loading and release characteristics.

**Controlled-Release Properties.**

The effectiveness of IA therapies depends largely on maintaining therapeutic concentrations within the joint over prolonged periods [21,22]. NDs are well suited to sustained release in vitro, as interactions between their functionalized surfaces and therapeutic molecules can enable release through diffusion, desorption, or degradation of surface coatings [94,97,98,103]. Release kinetics can be further tailored by incorporating NDs into hydrogels or polymeric matrices [103,104]. For example, adsorption of vascular endothelial growth factor (VEGF) onto NDs incorporated with a gelatin methacrylate hydrogel reduced burst release and prolonged VEGF delivery in vitro, while ND-reinforced hydrogels have also been shown to modulate MSC behavior in a bone-regeneration context [103,104]. However, these findings are derived from in vitro and non-cartilage regenerative models. Whether similar release profiles are maintained in synovial fluid, and whether NDs are retained within the joint or cleared through pathways similar to other nanoparticles remains to be established [79]. Sustained release may be particularly relevant for therapeutic peptides and growth factors that require sustained local exposure [98].

Potential Application in Cartilage Repair and OA Therapy

ND-based delivery systems remain at an early preclinical stage in OA and cartilage applications. In a rat model of OA, icariin-loaded NDs reduced inflammatory mediators, including IL-6 and TNF-α, decreased MMP-3 expression, and attenuated cartilage degradation [58]. These findings support potential anti-inflammatory and cartilage-protective effects but should not be interpreted as evidence of cartilage regeneration [105,106]. Beyond inflammation control, NDs have been investigated as carriers for regenerative molecules, such as growth factors, peptides, and other bioactive molecules in regenerative medicine [103,106]. However, much of this evidence originates from non-cartilage applications, and direct evidence demonstrating ND-mediated regeneration of articular cartilage remains limited. Their nanoscale dimensions, adaptable surface chemistry and controlled-release properties therefore provide a rationale for further investigation as IA delivery systems, although their cartilage penetration, tissue retention, and regenerative efficacy require further evaluation [97,106].

Challenges and Translation Considerations

Although the physicochemical properties of NDs support their investigation as IA carriers, important biological and translational questions remain unresolved. Their IA residence time, distribution between synovial and cartilage tissues, and routes of clearance have not been well characterized. Similarly, direct evidence demonstrating penetration of NDs into articular cartilage is currently limited. While the small primary particle size of NDs may favor tissue transport, aggregation in aqueous environments can substantially increase their effective particle size and may consequently restrict cartilage penetration [79,93]. Long-term safety following repeated IA administration also requires investigation, particularly because NDs are non-degradable and OA may require repeated or prolonged treatment [107,108]. Furthermore, evidence from large-animal models and human studies remains lacking.

Additional translational considerations include standardization of ND manufacturing and reproducibility of surface functionalization, as these factors can influence formulation characteristics, drug loading, and biological behavior [94]. Until these questions are addressed, NDs should be considered an emerging controlled-release platform rather than an established option for cartilage delivery. Future studies should therefore evaluate ND-based therapeutic release together with quantitative assessments of IA retention, cartilage penetration, biodistribution, clearance, and long-term safety, using approaches established for other cartilage-targeting carriers [28,29].

#### 3.3.3. Biomemtic and Surface-Engineered Nanoparticles

Cell Membrane-Coated Nanoparticles

Cell membrane-coated nanoparticles represent a recent advance in biomimetic drug delivery. By coating synthetic nanoparticles with membranes derived from macrophages, neutrophils, erythrocytes, or stem cells, these systems can evade immune recognition, prolong IA retention, and improve targeting of inflamed joint tissues [109]. Neutrophil membrane-coated nanoparticles have been shown to supress synovial inflammation and protect cartilage in experimental inflammatory arthritis [56]. Macrophage membrane-coated nanoparticles have shown enhanced accumulation within OA joints and greater anti-inflammatory efficacy in preclinical studies, highlighting their potential to increase therapeutic specificity while minimizing systemic exposure [110].

## 4. Emerging Intelligent Controlled-Release Systems and Future Perspectives

**Figure 4:**
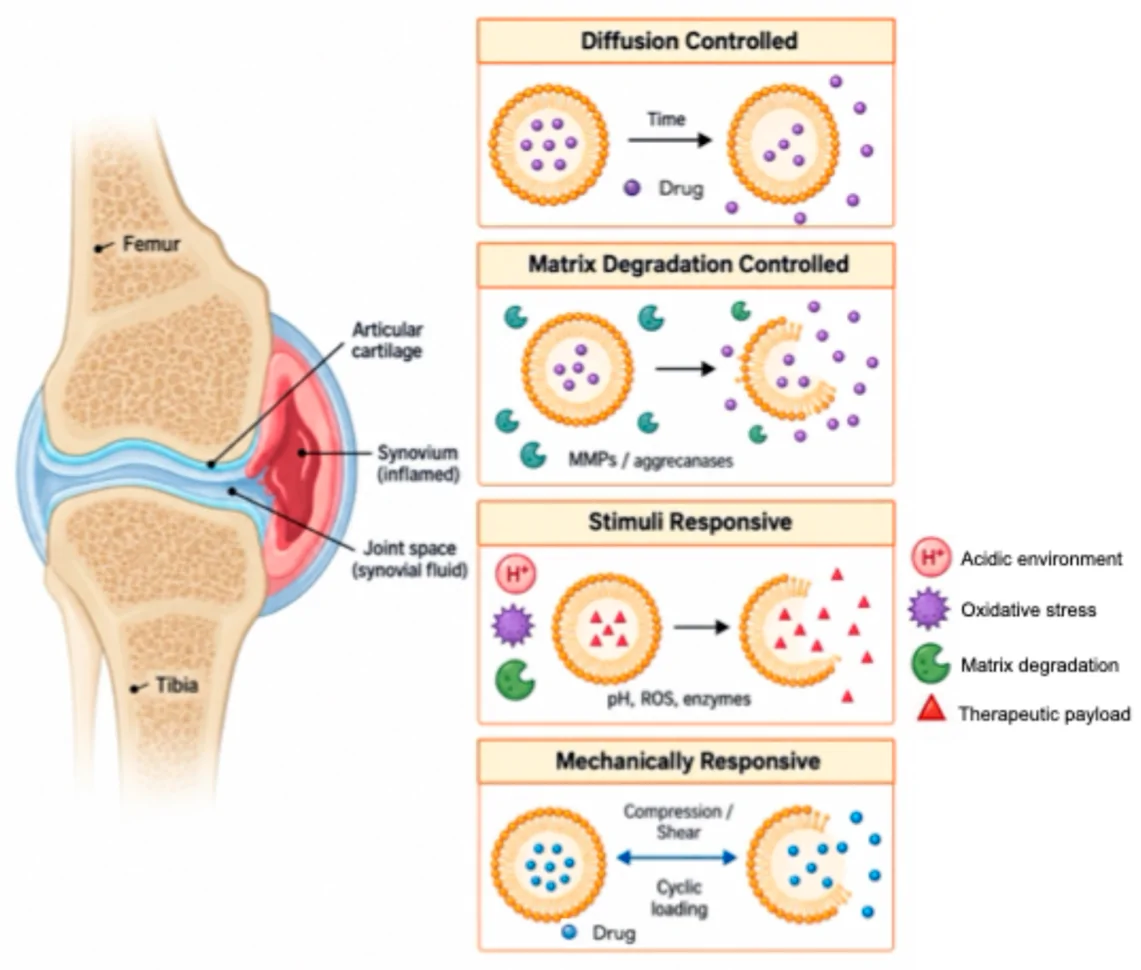
Intelligent controlled-release systems for cartilage regeneration. Schematic illustration of intelligent drug delivery platforms that exploit pathological features of the osteoarthritic joint, including acidic pH, elevated reactive oxygen species (ROS), increased matrix-degrading enzymes (MMPs, aggrecanases), and mechanical loading (compression/shear, cyclic loading). These stimuli trigger diffusion-controlled, degradation-controlled, stimuli-responsive, or mechanically responsive release of therapeutic agents, enabling localized and sustained drug delivery while reducing off-target effects. Collectively, these advanced platforms promote cartilage protection, reduce inflammation, and support cartilage regeneration.

While conventional controlled-release systems have significantly improved IA drug delivery, the next generation of regenerative therapies is increasingly focused on intelligent delivery platforms that respond dynamically to the pathological microenvironment of OA joints [19,52,73]. Unlike passive sustained-release systems, these “smart” platforms release therapeutic agents in response to specific biological, chemical, or mechanical stimuli, providing precise spatial and temporal control over drug delivery while minimizing systemic exposure and off-target effects (Figure 4) [19,71,73,111].

### 4.1. Stimuli-Responsive Delivery Systems

Several pathological features of the OA joint have been exploited to achieve stimuli-responsive therapeutic release [71,73]. The mildly acidic environment associated with inflammation has prompted the development of pH-responsive systems that selectively release therapeutic agents within diseased tissues while remaining relatively stable under physiological conditions [19]. Similarly, elevated levels of ROS have led to the development of ROS-responsive carriers capable of simultaneously scavenging free radicals and releasing anti-inflammatory or regenerative therapeutics in response to oxidative stress; cartilage-targeting hydrogel microspheres with ROS-responsive linkers, for example, reduced cartilage degeneration in a rat OA model [52,71,73]. MMPs and aggrecanases, key mediators of cartilage degradation, provide another attractive trigger for controlled drug delivery. Enzyme-responsive systems employ enzyme-cleavable linkers that selectively degrade within diseased tissue, enabling localized therapeutic release at sites of active tissue destruction [59,71,73]. Given the mechanically dynamic environment of synovial joints, mechanically responsive systems have also attracted increasing interest. These platforms can respond to compressive loading, shear stress, or cyclic joint movement, offering the potential to couple therapeutic activity with the mechanical environment of the joint [19,71]. Piezoelectric micro- and nanomaterials represent an emerging extension of this approach. When subjected to mechanical deformation, these materials can generate localized electrical signals that may influence cellular and tissue responses and have therefore been investigated for regenerative medicine applications. Piezoelectric materials can also be incorporated into composite or hydrogel-based systems and activated by physiological mechanical loading or externally applied stimuli such as ultrasound, providing opportunities for remotely or mechanically responsive therapeutic platforms [112]. However, their application to OA and cartilage-specific controlled delivery remains predominantly preclinical and requires further investigation.

### 4.2. Hybrid and Multifunctional Platforms

Recent advances have increasingly combined multiple delivery technologies to overcome the complex biological barriers associated with cartilage regeneration [19,71]. Hybrid systems incorporating nanoparticles within hydrogels, scaffold–nanoparticle composites, or biomimetic cell membrane-coated nanocarriers integrate the complementary advantages of multiple platforms into a single delivery system [19,22,60,85]. These multifunctional constructs can improve IA retention, enable sustained and stimuli-responsive drug release, enhance cartilage targeting, and simultaneously support tissue regeneration [61,71,73,85].

Beyond controlled drug delivery, next generation platforms are increasingly integrating diagnostic imaging agents, biosensors, gene-editing technologies, and cell-based therapies [105,106]. Such systems have the potential not only to deliver therapeutics but also to monitor disease progression, provide real-time feedback, and adapt treatment in response to changes within the joint microenvironment [19,105]. Together, these advances represent an important step toward precision regenerative medicine for OA [105,106].

## 5. Conclusions

AC regeneration remains a major clinical challenge because of the tissue’s limited intrinsic healing capacity, dense ECM, and the rapid clearance of therapeutics following IA administration. Although current pharmacological, surgical, and regenerative strategies can alleviate symptoms and promote tissue repair, their long-term efficacy remains limited by poor drug retention, inadequate tissue penetration, and insufficient therapeutic persistence within the joint.

Controlled-release delivery systems have emerged as promising approaches to overcome these limitations by enabling the localized and sustained delivery of therapeutic agents. Scaffolds, hydrogels, nanoparticles, and hybrid platforms each offer distinct advantages, while recent advances in biomaterial engineering have led to intelligent delivery systems capable of responding to pathological stimuli within the OA joint. Among emerging nanocarriers, NDs offer high drug-loading capacity, adaptable surface chemistry, and sustained-release properties. However, their application to articular cartilage remains predominantly preclinical, and cartilage-specific evidence is currently limited. Further studies are needed to determine whether these delivery characteristics translate into meaningful cartilage protection, disease modification, or regeneration. Despite encouraging preclinical progress, important challenges remain before these technologies can be translated into routine clinical practice. Continued advances in biomaterial design, manufacturing scalability, safety evaluation, and clinical validation will be essential for realizing the full potential of controlled-release systems. With ongoing interdisciplinary collaboration, these platforms have the potential to transform OA management by improving cartilage repair and providing more durable therapeutic options for patients with cartilage injury and degenerative joint disease.

## Figures and Tables

**Table 1:** Representative controlled-release strategies for overcoming intra-articular delivery barriers.

Delivery Platform	Representative Strategy	Main Barrier Addressed	IA Retention/Release	Cartilage Penetration/Targeting	Key Finding	Main Limitation	Ref.
Scaffold	TGF-β3-immobilized PLGA-based scaffold	Rapid clearance	Localized sustained delivery	Not evaluated	Supported ECM formation and cartilage repair	Requires implantation; preclinical evidence	[50]
Hydrogel	Thermosensitive hydrogel + chondrocyte exosomes	Rapid clearance	Sustained local delivery	Not quantified	Reduced inflammation and promoted cartilage repair	Long-term performance unknown	[51]
Responsive hydrogel microspheres	ROS-responsive, cartilage-targeted HA microspheres	Clearance; targeting; inflammatory microenvironment	Sustained, ROS-responsive release	Cartilage-targeted	Improved cartilage targeting and therapeutic response	Complex formulation; preclinical evidence	[52]
Polymeric nanoparticles	PLGA–resveratrol	Rapid clearance	Drug detected in joint tissues for >35 days	Not evaluated	Prolonged local exposure and reduced OA progression	Cartilage penetration not demonstrated	[53]
Lipid nanoparticles	FGF18 mRNA	Cargo instability; rapid clearance	Local expression for up to 96 h	Chondrocyte delivery demonstrated; depth not quantified	Promoted cartilage-related anabolic effects	Long-term IA fate unknown	[54]
Cationic nanocarrier	Avidin (~7 nm)	Poor penetration and retention	Approximately 1–2 weeks retention	Full-thickness penetration	Demonstrated charge-mediated cartilage uptake and retention	Binding must be balanced with tissue diffusion	[31]
Cationic polymeric nanocarrier	PAMAM–IGF-1 (<10 nm)	Poor penetration; rapid clearance	~10-fold longer joint half-life	Full-thickness penetration	Improved IGF-1 delivery and reduced cartilage degeneration	Requires optimization of size and charge	[29]
Cartilage-targeted nanoparticles	Collagen II-binding polymer NPs (~38 nm)	Poor targeting and retention	Enhanced cartilage retention	Up to 72-fold greater cartilage targeting vs. control	Demonstrated ligand-mediated cartilage targeting	Dependent on particle size and binding affinity	[30]
Inorganic nanoparticles	Cationic MnO_2_ NPs (<20 nm)	Poor penetration; oxidative stress	~75% retained at 1 week	Cartilage penetration demonstrated	Reduced oxidative stress and protected cartilage	Long-term clearance and safety uncertain	[55]
Biomimetic nanoparticles	Neutrophil membrane-coated NPs	Targeting; inflammatory microenvironment	Enhanced localization to inflamed joints	Cartilage penetration not quantified	Reduced synovitis and cartilage damage	Studied in inflammatory arthritis rather than OA	[56]
Carbon-based nanoparticles	Carbon dots–TNF-α siRNA	Cargo protection; inflammatory signaling	Not reported	Not quantified	Reduced inflammatory signaling and promoted chondrogenesis	Limited direct IA cartilage-delivery evidence	[57]
Nanodiamonds	Icariin–ND–icariin	Sustained drug delivery	Sustained ICA release up to 28 days in vitro; IA retention not measured	Not evaluated	Reduced inflammatory markers and cartilage degeneration	Limited cartilage-specific evidence	[58]
Enzyme-responsive system	MMP-2-responsive microgel	Rapid clearance; inflammatory microenvironment	Enzyme-triggered controlled drug release	Not evaluated	Reduced inflammation and attenuated OA progression	Preclinical evidence	[59]
Hybrid hydrogel–NP system	Hydrogel + PLGA nanoparticles	Rapid clearance	Prolonged local delivery	Not quantified	Sustained therapeutic activity	Increased formulation complexity	[60]
Dual-responsive nanohydrogel	Stimuli-responsive delivery	Clearance; poor penetration	Sustained IA release	Improved penetration reported	Combined sustained release and cartilage penetration	Recent preclinical evidence only	[61]

IA, intra-articular; ECM, extracellular matrix; HA, hyaluronic acid; NP, nanoparticle; PLGA, poly(lactic-co-glycolic acid); ROS, reactive oxygen species; ND, nanodiamond; OA, osteoarthritis. “Not evaluated” or “not quantified” indicates that the corresponding parameter was not directly assessed in the cited study.

## Data Availability

No new data were created or analyzed in this study. Data sharing is not applicable to this article.
